# Six-Year Implants Follow-Up After Guided Bone Regeneration Using Autologous Tooth Graft: Innovative Biomaterial for Bone Regeneration Tooth Transformer^®^

**DOI:** 10.3390/jfb16050172

**Published:** 2025-05-09

**Authors:** Elio Minetti, Angelo Michele Inchingolo, Laura Ferrante, Grazia Marinelli, Francesco Inchingolo, Alessio Danilo Inchingolo, Andrea Palermo, Gianna Dipalma

**Affiliations:** 1 Department of Biomedical, Surgical and Dental Science, University of Milan, 20122 Milan, Italy; elio.minetti@gmail.com; 2 Department of Interdisciplinary Medicine, University of Bari “Aldo Moro”, 70124 Bari, Italy; angeloinchingolo@gmail.com (A.M.I.); lauraferrante79@virgilio.it (L.F.); graziamarinelli@live.it (G.M.); alessiodanilo.inchingolo@uniba.it (A.D.I.); giannadipalma@tiscali.it (G.D.); 3 Department of Interdisciplinary Medicine, University of Salento, 73100 Lecce, Italy; andrea.palermo@unisalento.it

**Keywords:** autogenous dentin particulate, bone regeneration, dental biomaterials, grow factors, high-speed grinder, low-speed grinder, tooth graft, tooth transformer, autologous tooth, reconstructive surgical procedures, Tooth Transformer^®^

## Abstract

Objectives: Recently, there has been great interest in teeth and their derivatives as suitable substrates for the treatment of alveolar bone defects. This retrospective study evaluates the clinical and radiographic outcomes of implants inserted in a site that underwent GBR procedure using a tooth derivate material. Materials and methods: A total of 21 patients received a GBR using an autologous extracted tooth. Four months after the GBR techniques, the implants were inserted and were followed for an average of 5.28 + −1.10 years after loading. The X-ray was analyzed after a period of 63.36 + −13.2 months for a total follow-up period. Results: A total of 28 implants were inserted. All the implants were clinically functional after the follow-up period. The average bone loss from the X-ray images was 0.1208 + −0.1307. Conclusion: Within the limitations of this study, the use of a tooth as a graft using a tooth transformer device guarantees the production of bone and maintenance over time.

## 1. Introduction

Bone regeneration, particularly in dentistry, has experienced significant growth in recent decades, with biotechnology playing a crucial role [1,2,3,4].

Various bone substitutes, including allografts, xenografts, and autografts, have been proposed as post-extraction grafting materials to prevent periodontal defects. All studied biomaterials have shown the ability to reduce the bone resorption suffered in the buccal alveolar cortical bone after extraction. Autologous bone remains the gold standard due to its unique combination of osteoinductive, osteoconductive, and osteogenic properties. However, it has its own drawbacks, such as having limited obtainability in intraoral areas, requiring general anesthesia to be obtained from extraoral areas, causing an extra surgical trauma, and having a short resorption time [5,6,7,8,9,10]. Osteoinductive grafting materials stimulate bone formation by inducing the differentiation of multipotent mesenchymal stem cells from the surrounding host bone tissue [10,11,12,13]. Ongoing research is focused on developing biocompatible, cost-effective biomaterials that promote new bone formation with properties like natural bone, while minimizing morbidity and achieving optimal results in the shortest possible time. In recent years, teeth extracted for various reasons have increasingly been used as bone graft materials with high success rates, rather than being discarded as clinical waste. This shift is due to the striking structural similarities between teeth and bone. Both teeth and maxillofacial bones originate from the same neural crest cells and share a comparable composition of type I collagen and hydroxyapatite [14,15,16,17]. Additionally, dentin contains key growth factors such as Transforming Growth Factor-Beta (TGF-β), Bone Morphogenetic Proteins (BMPs), and insulin-like growth factors I and II, all of which play a crucial role in bone regeneration [18,19,20,21,22,23]. Additionally, it offers the advantage of not inducing host tissue reactivity or heterotopic bone formation, which is a crucial safety factor when selecting a graft [24,25,26]. Bone Morphogenetic Protein 2 (BMP-2) plays a crucial role in stimulating undifferentiated mesenchymal and osteoblastic cells, effectively promoting osteogenesis. Recent case reports have documented the successful use of an autologous, partially demineralized dentin matrix in various bone augmentation procedures, demonstrating significant clinical efficacy [27,28,29,30,31]. One of the most important evaluations in dentistry is post-extraction volumetric changes. These changes can hinder implant placement [32,33,34,35]. Numerous studies have evaluated the implant survival rate after 5 or 10 years. The implant survival rates range from 92.9 to 96–24% [36,37,38]. Some authors have instead analyzed peri-implant bone loss over time, estimating approximately 0.4 mm (±0.7) after 12 months [39,40,41]. The most interesting data for comparison with our study are those evaluating peri-implant bone loss in regenerated sites. The average values from these studies range from 2.48 mm (±0.80) to 3.45 mm (±0.63) [41,42,43]. A recent human study has demonstrated high histological and histomorphometrical levels of vital bone formation in GBR procedures. This was achieved using autologous demineralized dentin matrix grafts derived from freshly extracted teeth, processed with the innovative Tooth Transformer device [44,45,46].

A recent study has demonstrated the presence of BMP-2 in the graft material produced from the extracted tooth after treatment with the Tooth Transformer device (Tooth transformer srl Milan—Italy) [47].

The aim of the present study is to evaluate, after GBR therapies, the bone loss around 28 dental implants, from 21 patients, placed after regeneration procedures using an autologous graft derived from extracted teeth, with a follow-up period ranging from 5 to 7 years [48,49,50]. The objectives of the present study were also to evaluate the implant success rate, implant survival rate, and peri-marginal bone loss of implants placed in regenerated bone tissue using an extracted autologous tooth processed with the Tooth Transformer after 5 years [51,52].

## 2. Materials and Methods

### 2.1. Study Design and Patient Selection

This study is a retrospective chart review that includes 21 consecutive patients who underwent alveolar bone regeneration procedures using autologous tooth-derived grafts at a single clinic between 2017 and 2020. In total, 28 dental implants were inserted into 21 alveolar bone defects, as some sites required the placement of multiple implants. These patients were followed up for a period ranging from 4 to 5 years.

Inclusion Criteria

Patients included in the study (Table 1) were those who required dental implants after tooth extraction, which resulted in reduced bone volume necessitating regeneration through a Guided Bone Regeneration (GBR) procedure. Alveolar preservation or ridge preservation techniques were used to allow implant placement after hard tissue healing.

Exclusion Criteria

Patients were excluded if they were smokers, pregnant, or had systemic conditions such as diabetes, cancer, HIV, bone diseases, metabolic diseases, or if they were undergoing treatment with bisphosphonates, immunosuppressive agents, radiotherapy, or chemotherapy.

Surgical Procedures and Follow-Up

In all cases, the defects were covered with an OsseoGuard collagen membrane (Collagen Matrix, Oakland, NJ, USA). Before treatment, each patient underwent comprehensive radiographic evaluation using orthopantomograms (OPGs) and cone beam computed tomography (CBCT) to assess the extent of bone loss and defects.

Postoperative follow-up evaluations were carried out using intraoral radiographs and periodic clinical examinations at 3, 6, and 12 months, and then yearly. The implant survival rate and peri-implant bone loss were assessed by comparing radiographic images taken immediately after implant placement and during follow-ups.

Tooth Transformer Device

The Tooth Transformer device (Tooth Transformer Srl, Milan, Italy) was used to process extracted teeth into graft material. The device follows a standardized protocol, which involves thorough cleaning of extracted teeth to remove restorative materials, cement, and tartar. The teeth are then ground into optimally sized particles and undergo partial demineralization using solutions contained in a single-use kit. The kit includes six liquids organized in one box: one container with H₂O₂, one with HCl, and four with demineralized water [53,54,55,56,57,58,59,60]. This process, lasting approximately 25 min, preserves key growth factors naturally present in dentin, such as Bone Morphogenetic Protein-2 (BMP-2) and Transforming Growth Factor-Beta (TGF-β), ensuring the material retains its osteoinductive and osteoconductive properties, thus promoting bone regeneration and implant integration [61]. Inferential statistics, including paired t-tests or ANOVA, were used to compare bone loss between different time points and to assess correlations between patient demographics, implant type, and bone resorption rates.

Ethical Approval

The clinical study protocol was approved by the Ethics Committee of the University of Chieti on 21 March 2019 and was registered under the number 638—21/3/19.

### 2.2. Surgical Procedures

Before surgery, patients underwent clinical and radiographic examinations, including orthopantomograms (OPGs), intraoral radiographs, and cone beam computed tomography (CBCT) when necessary. One week prior to regeneration or implant surgery, all patients received a professional oral hygiene session [62,63]. On the day of surgery, local plexus anesthesia was administered using articaine with epinephrine to enhance hemostasis and reduce postoperative discomfort. Teeth were extracted using atraumatic techniques to preserve the surrounding alveolar bone; for complex extractions, piezosurgical instruments were utilized to minimize bone trauma. Immediately after extraction, teeth were thoroughly cleaned [64]. Any restorative materials (such as amalgam, composites, or endodontic sealers), cement, tartar, and residual periodontal ligament tissues were meticulously removed using a high-speed diamond bur under constant irrigation. Teeth were then sectioned into smaller fragments to facilitate the subsequent graft preparation process. The extracted tooth fragments were processed using the Tooth Transformer device (Tooth Transformer Srl, Milan, Italy).

#### 2.2.1. Tooth Transformer Device and Graft Preparation

The Tooth Transformer device is an automated system designed to transform autologous teeth into biocompatible graft material. It follows a standardized protocol that includes the following:

- Mechanical grinding of the tooth into particles of optimal size.
- Chemical decontamination and partial demineralization using a series of six solutions contained in a sterile, single-use kit (one container with hydrogen peroxide [H₂O₂], one with hydrochloric acid [HCl], and four with demineralized water).
- Preservation of key growth factors such as Bone Morphogenetic Protein-2 (BMP-2), Transforming Growth Factor-Beta (TGF-β), and Insulin-like Growth Factors (IGFs), essential for bone regeneration.

The entire process takes approximately 25 min, producing sterile, osteoconductive, and osteoinductive graft material ready for immediate clinical use.

Following preparation, the dentin graft material was mixed with the patient’s fresh autologous blood collected directly from the surgical site using sterile syringes. No additional anticoagulant systems were utilized. The blood was gently mixed with the dentin particles to enhance graft handling properties and improve biological integration.

#### 2.2.2. Grafting and Implant Procedures

**Figure 1:**
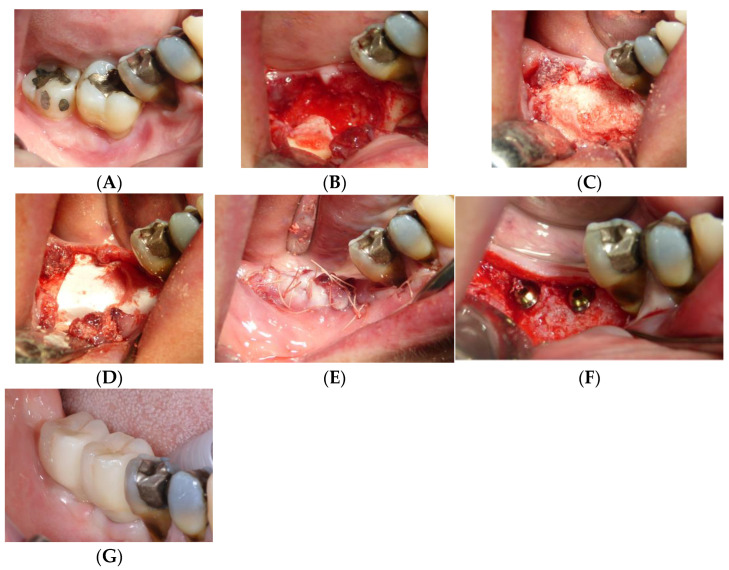
Operative sequence of a case, from extraction of two lower molars (**A**) followed by the regenerative procedure (**B**–**E**), insertion of the implants (**F**), and finally insertion of the prosthesis (**G**).

**Figure 2:**
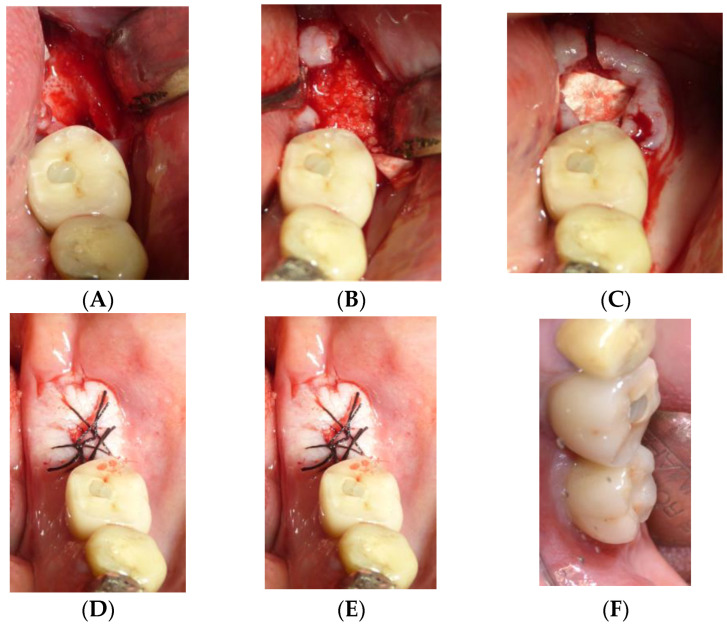
Operative sequence of another case, from the extraction of one lower molar (**A**) followed by the regenerative procedure (**B**–**D**), insertion of the implants (**E**), and finally the insertion of the prosthesis (**F**).

The dentin–blood mixture was placed into the alveolar defect, ensuring complete filling of the cavity. A resorbable collagen membrane (OsseoGuard, Collagen Matrix, Oakland, NJ, USA) was placed over the graft to stabilize the material and prevent soft tissue invasion during early healing. The flap was repositioned and sutured using non-resorbable sutures to achieve primary closure. Patients were prescribed postoperative antibiotics (Amoxicillin, 1 g twice daily for 7 days) and analgesics (Ibuprofen, 600 mg as needed) and instructed to rinse with chlorhexidine mouthwash to support healing. After a healing period of approximately 4 months, implant surgery was performed. A full-thickness flap was elevated, and implant site preparation was completed using a sequential drilling protocol to achieve primary stability. The implants were placed at the crestal bone level and allowed to osseointegrate before prosthetic loading. Final impressions were taken after implant healing to fabricate full ceramic prostheses (Figure 1 and Figure 2).

### 2.3. Follow-Up

Postoperative follow-up assessments were conducted at regular intervals to monitor the stability of the implants and evaluate the bone remodeling process. Patients were scheduled for clinical evaluations for 1 month, 3 months, 6 months, and then annually following implant placement. Each visit included a detailed assessment of soft tissue healing, implant stability, and potential signs of complications such as mucositis or peri-implantitis [65]. Radiographic evaluations, with Irys version 16.0 device (BioNano Genomics, San Diego, CA, USA), played a crucial role in tracking peri-implant bone changes over time [66,67,68,69,70,71]. Digital radiographic software, Mayray hyperion ×5, was employed to analyze bone levels, using the implant collar as a reference point to measure the distance between the implant shoulder and the mesial and distal marginal bone. In addition to conventional radiographs, some cases required three-dimensional imaging with cone beam computed tomography (CBCT) to assess the quality of bone regeneration and verify implant osseointegration [72]. The CBCT scans provided a more comprehensive view of bone density and volumetric changes, particularly in cases where significant ridge augmentation was performed. The stability of the peri-implant bone was evaluated by comparing radiographs taken immediately post-implant placement to those taken at subsequent follow-ups. Any marginal bone loss was recorded and analyzed to determine trends over time [73,74].

### 2.4. Statistical Analysis

The objectives of this study were to evaluate the implant success rate, implant survival rate, and peri-marginal bone loss after 5 years. The collected data were statistically processed to assess the effectiveness of the Tooth Transformer-derived graft in preserving alveolar bone integrity over the long term.

Descriptive statistics were used to calculate mean values, standard deviations, and ranges for each variable, including peri-implant bone loss, implant success rate, and other clinical outcomes [51,52,75,76,77,78,79,80,81].

A *p*-value < 0.05 was considered statistically significant [82,83].

The statistical analysis was performed using SPSS software (version X) [48,50,84,85,86,87,88,89,90,91].

## 3. Results

The sample analyzed consisted of 21 patients and 28 placed implants. The implant success rate was 100% four years after the implant placement phase. Retrospective radiographic evaluations performed on periapical radiographs measured mesial–distal bone levels around dental implants over time. The data indicated that the average mesial bone loss was 0.14302 + −0.0107 and distal bone loss was 0.09934 + −0.0143. The mean peri-implant bone loss, as measured from the radiographic follow-up, was 0.1208 ± 0.1307 mm. This represents a significantly lower resorption rate compared to other studies evaluating implants placed in regenerated bone, where bone loss values ranged from 0.4 mm to 1.6 mm within the first year. A breakdown of the peri-implant bone loss measurements showed that the average mesial bone loss was 0.14302 ± 0.0107 mm, while the distal bone loss was 0.09934 ± 0.0143 mm [92]. These minimal values indicate high stability of the regenerated bone. The reduced resorption may be attributed to the biocompatibility of dentin-based grafts, their slow resorption rate, and their ability to retain bioactive molecules that promote bone remodeling. No signs of inflammation, necrosis, or foreign body reaction were observed at any time point, confirming the high biocompatibility of the autologous dentin graft.

**Figure 3:**
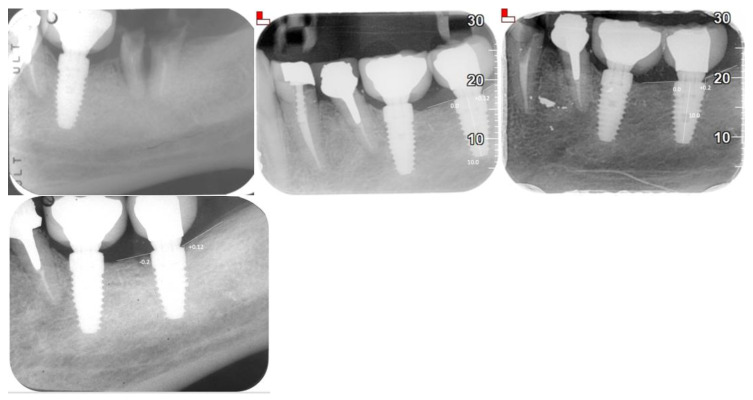
X-ray follow-up during the time. Extractions from 2021, 2022, and 2024.

**Figure 4:**
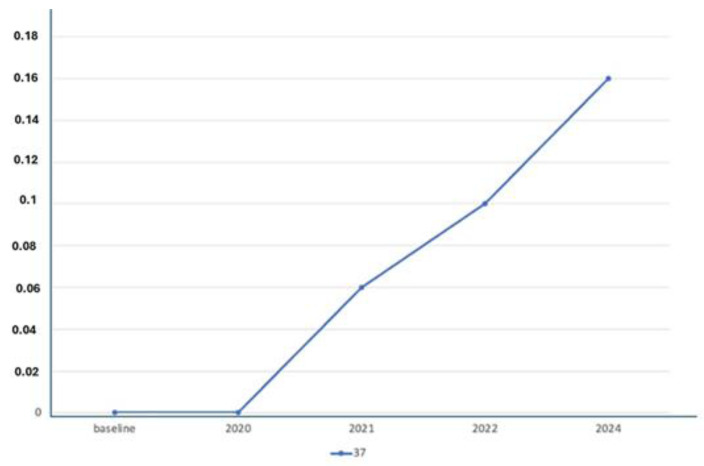
Average resorption of the patient from Figure 3.

**Figure 5:**
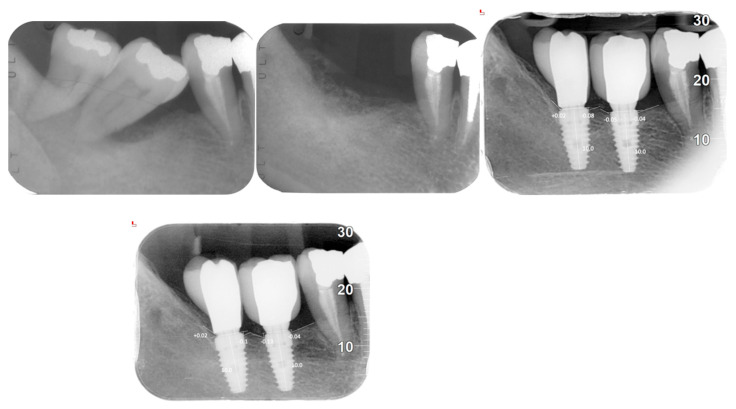
X-ray follow-up during the time. Extractions from 2019, 2021, and 2023.

**Figure 6:**
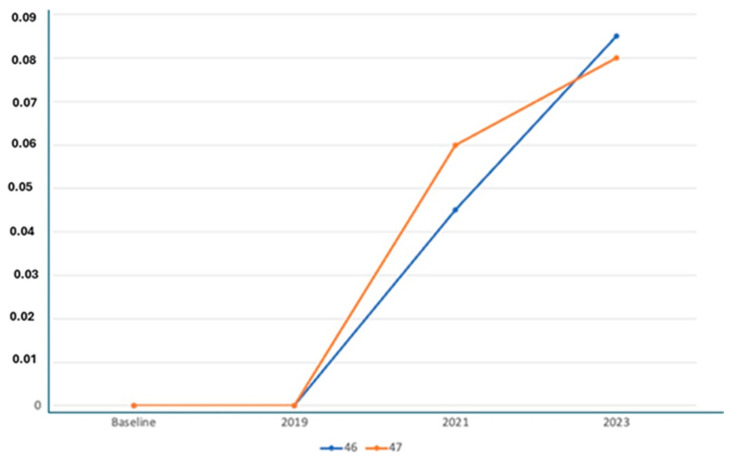
Average resorption of the patient from Figure 4.

X-ray Follow-ups. As part of the follow-up, X-rays were taken at different time points for the patients to assess the progression of bone resorption and implant stability. Figure 3 shows the X-ray follow-up during the time, showing the extraction and X-ray results from 2021, 2022, and 2024. Figure 4 provides a summary of the average resorption seen in the patient depicted in Figure 3. Figure 5 shows the X-ray follow-up over time, showing the extraction and X-ray results from 2019, 2021, and 2023. Figure 6 summarizes the average resorption observed in the patient depicted in Figure 5 [93].

The following tables, Table 2 and Table 3, provide an overview of the patient demographics and implant data used in this study.

## 4. Discussion

The present study demonstrated a high degree of stability in regenerated bone using dentin, which supported the analyzed implants throughout the entire follow-up period. Histological analysis was performed on bone core biopsies obtained at different time points post-grafting (3, 6, and 12 months). Hematoxylin and eosin (H&E) staining, along with Masson’s trichrome staining, were used to evaluate the structural integration of the dentin graft within the host bone. At 3 months, early signs of new bone formation were visible, with osteoblasts actively depositing osteoid matrix along the surfaces of the dentin granules. Trabecular bone infiltration into the grafted area was evident, indicating the initiation of osteoconduction [94]. At 6 months, histological images revealed an intimate connection between the newly formed bone and the dentin particles, with clear evidence of bone remodeling activity. Osteoclasts were occasionally observed on the dentin surfaces, confirming that the material was undergoing progressive resorption and replacement with new bone. At 12 months, the graft material was almost completely integrated, with mature lamellar bone structures replacing most of the dentin matrix [95]. The amount of bone loss was minimal, which aligns well with findings in the existing literature on the topic [96,97,98]. This is particularly noteworthy considering that bone resorption is one of the most common challenges in implantology. Moreover, the platform-switching technique applied in wide-diameter implants undoubtedly played a critical role in stabilizing the bone and reducing peri-implant bone loss. At the end of the 5-year follow-up, the cumulative implant survival rate was an impressive 100%. In reviewing the literature on implants placed in regenerated sites, the implant survival rate typically ranges between 97% and 100%, with most studies reporting results above 90%. These figures are quite comparable to those observed with implant bones placed in native bone, suggesting that regenerated tissue can offer similar outcomes to native bone in terms of implant survival and follow-up [99,100,101]. This finding supports the clinical application of regenerative techniques, such as the use of dentin grafts, in improving the stability and longevity of dental implants. The classification of defects is another important factor in predicting outcomes. For large defects, a slow-resorbing biomaterial, typically covered with a membrane, has been recommended for optimal healing [102]. This is consistent with the findings of other studies, such as a longitudinal study on bone resorption in implants placed in native bone, where the average resorption value was reported as 1.3 mm. These results underscore the importance of using suitable graft materials and techniques tailored to the size and nature of the defect. Furthermore, a prospective study examining 126 implants placed in defects treated with a Guided Bone Regeneration (GBR) procedure, using xenogeneic material and non-resorbable membranes, showed a 5-year survival rate of 93.1%. While this survival rate is slightly lower than the 100% reported in the current study, it reflects older techniques and materials, highlighting the significant progress made in regenerative implant procedures over the years. Additionally, a randomized study comparing different xenogeneic materials found no significant differences between the two materials under study [103]. After six months of loading, the average peri-implant bone loss at three years was 1.61 mm for the first group and 1.02 mm for the second group, further suggesting that both material types are effective in bone regeneration. Both resorbable and non-resorbable GBR techniques are proven methods that offer high stability for bone tissue, even over extended periods of time [104]. In Naenni’s research, for example, the bone tissue remodeling around implants was 0.23 mm (±0.46) for resorbable membranes and 0.17 mm (±0.28) for non-resorbable membranes, demonstrating minimal bone resorption even at the 5-year mark. These findings suggest that GBR techniques can provide predictable, long-term outcomes in implantology, contributing to the overall success and stability of implants. In a comprehensive analysis conducted in 2021, a total of 483 implants placed after GBR procedures were evaluated. After 12 months of loading, the failure rate was relatively low, with only 10 implants failing (2.3%), and a success rate of 98.2%. The radiographic results indicated an average bone resorption of 0.37 mm (±0.68), with medial resorption at 0.43 mm (±0.83) and distal resorption at 0.23 mm (±0.38). These figures are consistent with the current study’s results, indicating that the use of GBR techniques, particularly when combined with autologous grafts such as dentin, offers stable and predictable outcomes over time [105]. Dentin, being a mineralized tissue like bone, resorbs more slowly than traditional bone chips, ensuring a greater level of osteoconductive stability over time. As highlighted in our histological study, the presence of autologous proteins within dentin, which are homologous to those found in bone tissue, promotes a high level of regeneration. This feature is particularly advantageous as it enhances the integration of the graft with the surrounding bone, minimizing the risk of graft failure. These findings underscore the potential of autologous dentin grafts as a reliable alternative to conventional xenografts and allografts in dental implantology. In addition to their biocompatibility, dentin-based grafts offer the advantage of gradual resorption, which allows for long-term bone regeneration [106]. This characteristic makes them superior to synthetic graft materials, which often carry the risk of immune reactions or slower remodeling. Furthermore, the use of dentin derived from the patient’s own teeth reduces the likelihood of graft rejection, providing an added layer of safety for patients.

Despite the promising results presented in this study, it is important to acknowledge several limitations. One significant limitation is the relatively small sample size of only 21 patients [107,108]. While this sample size allowed for valuable insights, it may not be large enough to detect small differences in implant survival or peri-implant bone loss. Future research involving larger cohorts and multi-center trials would help to confirm these findings and provide more robust data. Additionally, while the follow-up period of up to 5 years is valuable, a longer observation period, such as 10 years, would be necessary to fully assess the long-term stability of the graft material and implants. Moreover, the study did not address the cost effectiveness of using autologous dentin grafts compared to other commonly used bone grafting materials [109,110,111,112,113,114,115]. This is an important consideration for clinical adoption, as the financial implications of using autologous grafts may influence their widespread implementation. Future studies should explore the economic aspects of dentin-based grafts and assess their viability from a cost–benefit perspective. In conclusion, this study highlights the clinical potential of autologous tooth-derived grafts in alveolar bone regeneration [116,117]. The high biocompatibility and osteoconductivity of dentin, combined with its gradual resorption, make it a promising alternative to synthetic or allogenic bone graft materials [106,118,119]. Given the low rate of peri-implant bone resorption observed in this study, the use of dentin-based grafts can be considered a reliable and cost-effective solution for bone regeneration. Clinically, this technique shows great promise in a variety of implant procedures, including socket preservation, sinus lift, and ridge augmentation. As future research further investigates the molecular mechanisms underlying dentin-mediated bone regeneration, including the potential role of growth factors like BMPs, VEGF, and FGF, the scope of applications for this innovative technique is likely to expand [120]. Results from the radiographic follow-ups confirmed that the average peri-implant bone loss remained minimal throughout the 5-year observation period, with mesial and distal bone levels demonstrating high stability. The consistency of these findings with previous studies on autologous dentin grafts suggests that this approach provides a reliable solution for maintaining peri-implant bone health [121].

Scanning electron microscopy (SEM) and energy-dispersive X-ray spectroscopy (EDS) analysis provided further insights into the microstructural composition of the grafted site. SEM images revealed a dense, interconnected bone network, while EDS confirmed the presence of calcium and phosphorus in ratios like natural bone. These findings highlight the osteoconductive and osteoinductive properties of the Tooth Transformer-processed dentin graft, supporting its long-term role in bone regeneration. This study confirms previous findings that tooth-derived grafts, particularly when processed with the Tooth Transformer device, provide a reliable alternative to traditional bone grafting materials. Additionally, the platform-switching technique used for wide-diameter implants may have further contributed to the preservation of marginal bone levels [122].

The integration of advanced technologies such as digital dentistry and 3D bioprinting could further enhance the outcomes of dentin-based bone augmentation procedures, paving the way for a new era of personalized regenerative dentistry [123,124].

## 5. Conclusions

This study highlights the viability of autologous tooth-derived grafts for alveolar bone regeneration, suggesting that they could serve as a reliable alternative to traditional graft materials. The findings show minimal bone resorption and excellent implant success over a 5-year period. However, further studies with larger sample sizes are needed to better understand the long-term impact of this innovative regenerative technique using demineralized teeth in oral bone regeneration procedures. The high biocompatibility of autologous tooth-derived grafts is clearly demonstrated by the stability of bone regeneration and the low rate of peri-implant bone resorption observed. The high survival rate of dental implants five years after loading further confirms the potential of tooth-derived grafts in supporting intraoral bone maintenance, preservation, and augmentation.

## Figures and Tables

**Table 1:** Between 2019 and 2021, twenty-one patients were treated with GBR using autologous extracted teeth. Twenty-height implants were inserted and after the healing period they were loaded with prosthesis.

Sample size	21 patients (7 male–14 female)
Average age	63 years (range 50–87)
Total extracted teeth	28 Incisive 0 Canine 1 Premolars 6 Molars 21
Reason to extraction	20 teeth were removed for periodontal reasons 5 teeth were removed for fractures 3 teeth were removed for caries
Type of teeth extracted	17 teeth were whole 11 teeth were endedontical
Dental implants	28 implants
Average implant lenght	10.72 mm (+−1.12)
Implants dimensions	25 diameter 5–4 diameter 4.5–1 diameter 4

**Table 2:** Summary of patient demographics and implant data.

Category	Details
Study Design	Retrospective chart review
Study Period	2017–2020
Number of Patients	21 (7 males, 14 females)
Average Age	63 years (range: 50–87)
Total Extracted Teeth	28
Tooth Types Extracted	1 canine, 6 premolars, 21 molars
Number of Implants Placed	28
Implant Dimensions	Diameter: 25 implants (5 mm), 4 implants (4.5 mm), 1 implant (4 mm)
Average Implant Length	10.72 mm (±1.12)
Bone Graft Material	Autologous dentin graft processed with the Tooth Transformer
Follow-up Period	4–5 years
Evaluation Methods	Orthopantomograms (OPG), cone beam computed tomography (CBCT), intraoral radiographs, clinical examinations
Follow-up Intervals	3, 6, 12 months and annually
Implant Success Rate	100% after 4 years
Peri-implant Bone Loss	Mean: 0.1208 mm (±0.1307)
Mesial Bone Loss	0.14302 mm (±0.0107)
Distal Bone Loss	0.09934 mm (±0.0143)
Key Outcomes	High stability of regenerated bone, minimal resorption, no inflammation or foreign body reaction
Statistical Analysis	Descriptive statistics, paired t-tests, ANOVA (*p* < 0.05)
Number of Implants per Year	2019: 13, 2020: 7, 2021: 8

**Table 3:** Summarized achievements.

Peri-Implant Bone Loss	
AVERAGE BONE LOSS	0.1208 + −0.1307
Mesial bone loss	0.14302 + −0.0107
Distal bone loss	0.09934 + −0.0143
Number of implants per year	
2019	13
2020	7
2021	8

## Data Availability

The original contributions presented in the study are included in the article, and further inquiries can be directed to the corresponding author.

## Abbreviations

Abbreviation	Full Term
BMP-2	Bone Morphogenetic Protein 2
CBCT	Cone Beam Computed Tomography
CEA	Not explicitly defined in the text (possibly a type of implant or specific protocol)
EDS	Energy-dispersive X-ray Spectroscopy
FGF	Fibroblast Growth Factor
g	gram
GBR	Guided Bone Regeneration
HCl	Hydrochloric acid
H&E	Hematoxylin and Eosin
HIV	Human Immunodeficiency Virus
H_2_O_2_	Hydrogen peroxide
N-RES	Non-Resorbable
OPGs	Orthopantomograms
PRF	Platelet-Rich Fibrin
RES	Resorbable
SEM	Scanning Electron Microscopy
SPSS	Statistical Package for the Social Sciences
TGF-β	Transforming Growth Factor-Beta
VEGF	Vascular Endothelial Growth Factor
X-ray	Radiograph
